# TMAO is involved in sleep deprivation-induced cognitive dysfunction through regulating astrocytic cholesterol metabolism via SREBP2

**DOI:** 10.3389/fnmol.2024.1499591

**Published:** 2024-11-28

**Authors:** Shan Zhu, Yue Wang, Yansong Li, Na Li, Yige Zheng, Qiao Li, Hongyan Guo, Jianyu Sun, Qian Zhai, Yaomin Zhu

**Affiliations:** ^1^Department of Anesthesiology and Center for Brain Science, The First Affiliated Hospital of Xi’an Jiaotong University, Xi’an, Shaanxi, China; ^2^Department of Anesthesiology, The Second Affiliated Hospital of Xi’an Jiaotong University, Xi’an, Shaanxi, China; ^3^The Second Clinical Medical College, Shaanxi University of Chinese Medicine, Xianyang, Shaanxi, China

**Keywords:** sleep deprivation, FMO3, TMAO, SREBP2, astrocytes, cholesterol metabolism

## Abstract

Sleep deprivation (SD) contributes to cognitive impairment. Astrocytic cholesterol biosynthesis is crucial for brain cholesterol homeostasis and cognitive function. However, the underlying mechanism of astrocytic cholesterol metabolism in SD-induced cognitive impairment has not been fully explored. Trimethylamine N-oxide (TMAO), a product of liver flavin-containing monooxygenase-3 (FMO3), has been shown to be increased in the urine of sleep-deprived humans and implicated with peripheral cholesterol metabolism. Nevertheless, how TMAO affects brain cholesterol metabolism remains unclear. In our study, increased FMO3 and brain TMAO levels were observed in the SD mice, and elevated levels of TMAO were confirmed to lead to SD-induced cognitive dysfunction. In addition, we found that the expression of sterol regulatory element-binding protein 2 (SREBP2) is decreased in the brain of SD mice, resulting in the reduction in brain cholesterol content, which in turn causes synaptic damage. Moreover, we demonstrated that TMAO inhibits the expression of SREBP2. In contrast, FMO3 inhibitor 3,3′-diindolylmethane (DIM) alleviates SD-induced cognitive impairment by targeting the liver–brain axis. In conclusion, our study revealed that the TMAO pathway is involved in memory impairment in SD mice through deregulating astrocytic cholesterol metabolism.

## 1 Introduction

Sleep loss appears common daily and has become a global public health issue. According to the World Health Organization, more than one-third of the world’s population suffers from sleep deprivation (SD; Ried et al., 2023; Ohayon et al., 2019). Sleep deprivation is closely associated with cognitive deficits, metabolic disorders, and even neurodegenerative diseases (Zhang M. et al., 2023; Huang et al., 2022; Lv et al., 2022). It is worth noting that synaptic plasticity is critical for learning and memory (Takeuchi et al., 2014). A recent study showed that defective working memory with decreased synaptic density and dendritic spines was detected in sleep-deprived mice (Tan et al., 2023). Therefore, there is strong evidence demonstrating that synaptic plasticity is closely related to the recovery and improvement of cognitive impairment after chronic sleep restriction (CSR).

The brain possesses the highest concentration of cholesterol among all organs, and its cholesterol metabolism is mainly synthesized locally because of the blood–brain barrier (BBB; Zhang and Liu, 2015; Björkhem and Meaney, 2004). It has been suggested that cholesterol metabolism is essential for synapse formation, vesicle formation, and membrane fluidity (Ali Moussa et al., 2023; Goritz et al., 2005). In addition, in adults, astrocytes are principally in charge of producing cholesterol, which is crucial for preserving cholesterol homeostasis and cognitive function by delivering cholesterol to neurons (Li et al., 2022). Studies have shown that disruption of the astrocytic cholesterol synthesis pathway can lead to axonal growth restriction, synaptic damage, and learning and memory impairment (Ferris et al., 2017; Xu et al., 2022). Loss of SREBP2, a major transcription factor regulating cholesterol biosynthesis, results in dysregulation of brain cholesterol homeostasis, which can contribute to impaired neurite growth and defective learning and memory (Ferris et al., 2017; Santello et al., 2019). Of note, defect in SREBP2 is linked to several neurodegenerative diseases, such as Huntington’s disease (HD) and Parkinson’s disease (PD; Suzuki et al., 2010; Birolini et al., 2021; Tang et al., 2023). However, whether astrocytic SREBP2-related brain cholesterol metabolism is involved in cognitive impairment after SD has not been reported.

Trimethylamine N-oxide (TMAO), a hepatic metabolite, generates from dietary factors and converted by the gut microbes to trimethylamine (TMA), followed by hepatic FMO3 conversion into TMAO (Wang et al., 2011; Koeth et al., 2013). The previous study has shown that TMAO can penetrate the BBB (Del Rio et al., 2017). Interestingly, increased levels of TMAO were observed in the plasma and brain of both Alzheimer’s disease (AD) and diabetic mice (Vogt et al., 2018; Govindarajulu et al., 2020). TMAO is closely related to sleep. In obstructive sleep apnea (OSA), a sleep-related disease, the elevated level of TMAO is strongly associated with OSA-related cardiovascular disease (Badran et al., 2023; Conotte et al., 2018; Liu et al., 2019). In addition, TMAO has been not only linked to the onset of age-related cognitive decline but also increased in the urine of sleep-deprived humans (Li et al., 2018; Giskeødegård et al., 2015). In addition, the previous research results of our group showed that SD led to the decrease of *Akkermansia muciniphila* (AKK) level in mice (Li N. et al., 2023), while AKK can reduce the concentration of TMAO (Chen et al., 2016; Wang et al., 2022), suggesting the possibility of increasing TMAO induced by SD. Nevertheless, the involvement of TMAO contributing to SD-induced cognitive deficit has not been investigated. Meanwhile, the TMA/FMO3/TMAO pathway is related to peripheral cholesterol metabolism (Warrier et al., 2015). One study has revealed that taking TMAO supplements reduced peripheral reverse cholesterol transport (Janeiro et al., 2018). However, to date, it has not explored the relationship between TMAO and cholesterol metabolism in the brain.

In this study, we hypothesized that FMO3/TMAO signaling causes altered synaptic plasticity by inhibiting astrocytic cholesterol metabolism resulting in memory impairment in CSR mice. We assessed the effect of TMAO on the expression of SREBP2. Our findings strongly supported that FMO3 inhibitor 3,3′-diindolylmethane (DIM), one of the key constituents of cruciferous vegetables (Sarkar et al., 2024), alleviates CSR-induced cognitive impairment by reducing TMAO level, upregulating astrocytic SREBP2 expression and brain cholesterol level, and finally reversing synaptic damage.

## 2 Materials and methods

### 2.1 Ethics approval and animal preparation

In this study, male C57BL/6 mice were housed 5/cage under controlled conditions with a 12-h light/dark cycle in an ambient temperature (23 ± 2°C) and humidity (55 ± 2%) controlled room. Water and food were provided *ad libitum*. All animal experiments were performed in accordance with the Xi’an Jiaotong University Guidelines for Animal Experimentation and were approved by the Animal Care and Use Committee of Xi’an Jiaotong University (Xi’an, Shaanxi, China).

### 2.2 Chronic sleep restriction

The CSR protocol was implemented according to our previous study (Zhang S. et al., 2023). 9- to 10-week-old (*n* = 78) mice were placed in the automated cylindrical apparatus (KW-BD, NJKEWBIO, Nanjing, China) with a spinning bar at approximately 7 rpm to consistently disrupt their sleep for 20 h (from 8 p.m. to 4 p.m.) for consecutive 1 week. Non-sleep-deprived mice were housed in the same apparatus without the spinning bar and served as controls (CON).

### 2.3 Grouping

#### 2.3.1 Validation of the role of TMAO in brain cholesterol metabolism

Mice (5–6 weeks old, *n* = 96) were fed a standard chow diet with or without 0.12% TMAO supplementation for 4 weeks (Chen et al., 2019); then, the CSR protocol was implemented, and mice were randomly divided into four groups: Control, TMAO, CSR, and CSR + TMAO. The diet of each group continued until the end of all experiments.

#### 2.3.2 Evaluation of the neurological function during CSR after modulating FMO3 expression

Mice (5–6 weeks old, *n* = 68) were fed a standard chow diet with or without 0.25% DIM supplementation for 4 weeks (Chen et al., 2019); then, the CSR protocol was implemented, and mice were randomly divided into four groups: Control, DIM, CSR, and CSR + DIM. The diet of each group continued until the end of all experiments.

### 2.4 Behavioral tests

Memory was evaluated using the novel object recognition (NOR; Lueptow, 2017) and the Y-maze test (Kraeuter et al., 2019) in mice at 10–11 weeks (*n* = 160). In the training session of NOR, the mice were allowed to explore freely in the apparatus with two identical objects for 10 min to form object memory. The testing session was performed 60 min after training. During the testing session, one identical object was swapped out for a new one and the mice freely explored the objects for 10 min. The discrimination index was calculated by dividing the time spent examining the novel object by the total time spent exploring both objects.

In the Y-maze test, mice were allowed to explore freely for 8 min. When the mouse entered the arm with all four paws, it was considered as an arm entry. Consecutive entries into all three arms were deemed to constitute an alternation. Accordingly, the percentage of alternation behavior was measured according to the following formula: % Alternation = (total number of alternations / total number of arms traversed −2) × 100%.

### 2.5 Primary astrocyte culture and stimulation

At a young age of 1–2 days, C57BL/6 mice’s cerebral cortices were harvested to get primary cortical astrocytes. Cells were seeded in a T75 flask containing 10 mL of medium (90% DMEM, 10% FBS, and 1% penicillin/streptomycin). To obtain mouse primary astrocytes, two mouse cortices were used per T75 flask preparation and then shaken for 2 h at 250 rpm using an orbital shaker to eliminate most of the microglia. Subsequently, the flask was transferred to an incubator. Cells from independent experiments at 80% confluence were stimulated with lipopolysaccharide (LPS, 100 ng/mL, Sigma-Aldrich, United States; Zhang S. et al., 2023) or TMAO (100 μM, Sigma-Aldrich, United States; Brunt et al., 2021) for 24 h.

### 2.6 Western blot analysis

Hippocampal tissue was subjected to RIPA buffer supplemented with protease inhibitors. The samples underwent electrophoresis on a 15% SDS-PAGE gel and were subsequently transferred to PVDF membranes. The membranes were incubated with the primary antibody overnight at 4°C after being blocked with PBS containing 5% non-fat dry milk for 2 h at room temperature and were then incubated overnight at 4°C with different primary antibodies including FMO3 (Abcam, ab126711), SREBP2 (ImmunoWay, YN0037), PSD95 (Cell Signaling, 3,450), synaptophysin (Synaptic Systems, 101,011), and ACTB (Abclonal, AC026). Following three washes with TBST, the membranes were subjected to peroxidase-conjugated secondary antibodies (goat anti-rabbit; goat anti-mouse) for 60 min at room temperature. The membrane bands were discovered using an ECL kit (Affinity, Shanghai, China), and the protein band densities were quantified using ImageJ. ACTB was used as an internal control.

### 2.7 Immunofluorescence staining

The mice were deeply anesthetized and transcardially perfused with ice-cold saline and 4% paraformaldehyde (PFA). Then, the entire brain was fixed with 4% PFA for 6 h, followed by a 30% sucrose solution to dehydrate. Coronal sections of the hippocampus (12-μm thickness) were collected using cryostat instruments. For immunofluorescence staining, the sections underwent three washes with PBS, blocked and perforated for 2 h at room temperature with PBS containing 0.3% Triton X-100 and 5% donkey serum, and then incubated with primary antibodies including GFAP (GeneTex, GTX85454), SREBP2 (SAB, 53501), PSD95 (Abcam, ab238135), and SYP (Synaptic Systems, 101,011) overnight at 4°C. Following three 10 min washes with PBS (pH 7.4), the sections were incubated for 2 h at room temperature with fluorescent dye-conjugated secondary antibodies (donkey anti-chicken Alexa Fluor 488; donkey anti-rabbit Alexa Fluor 594; donkey anti-mouse Alexa Fluor 488) in the dark. Finally, the sections were incubated with DAPI for 10 min, followed by three washes with PBS as described above. All confocal images were acquired using a confocal microscope (Olympus FV3000).

For primary astrocytes, cells were seeded in the confocal dish. Astrocytes were fixed in 4% paraformaldehyde for 10 min at 4°C. Subsequently, astrocytes were washed with PBS, blocked for 30 min at 37°C in blocking buffer, and then incubated with primary antibodies GFAP (GeneTex, GTX85454) and SREBP2 (SAB, 53501) overnight at 4°C. The next day, cells in confocal dishes were washed three times with PBS and incubated with secondary antibodies (donkey anti-chicken Alexa Fluor 488; donkey anti-rabbit Alexa Fluor 594) for 2 h at room temperature in the dark. The rest of the procedure is the same as for tissue immunofluorescence staining.

### 2.8 Quantitative RT-PCR

One side of the hippocampus was used for real-time PCR assays. Total RNA was extracted using a TRIzol reagent (Invitrogen, Carlsbad, United States) and reverse-transcribed into cDNAs using Hifair II 1st Strand cDNA Synthesis SuperMix for qPCR (Yeasen Biotechnology, Shanghai, China). The expression of the target gene was normalized to actin and quantified by the comparative cycle threshold method (2^−ΔΔCT^). The primer sequences were as follows: FMO3 forward: 5’-CCCACATGCTTTGAGAGGAG-3′, reverse: 5’-GGAAGAGTTGGTGAAGACCG-3′; SREBP2 forward: 5’-GCCTCTCCTTTAACCCCTTG-3′, reverse: 5’-CCAGTCAAACCAGCCCCCAG-3′.

### 2.9 Transmission electron microscopy

The hippocampus was trimmed to small pieces (1 mm^3^) and then was fixed in 2.5% glutaraldehyde in PBS and postfixed in 1% OsO4 at 4°C. The samples were then washed, dehydrated with a graded series of alcohol, incubated in 1% osmium tetroxide for 1 h at room temperature, and embedded in the medium, followed by ultrathin (80 nm) sectioning with an ultramicrotome. Electron micrographs of the hippocampal CA1 region were obtained utilizing a HITACHI electron microscope (HITACHI, H7650, Japan). Ten electron micrographs of each section were randomly taken from each field of view. The density of presynaptic vesicles, PSD length, width, and synaptic cleft width were quantified.

### 2.10 Golgi staining and image analysis

Mice were sacrificed immediately, and the brain tissues were immersed in the fixative (Servicebio, G1101) for more than 48 h. After that, the brain tissues were transferred into a Golgi-Cox staining solution (Servicebio, G1069) and kept in the dark for 14 days. Then, the brain tissues were immersed in distilled water 3 times and immersed in 80% glacial acetic acid overnight to become soft, and then placed in 30% sucrose. The brain sections were cut into 100 μm with a vibratome, pasted on a gelatin slide, and dried in the dark overnight. Images from hippocampal regions were collected using a confocal microscope (Nikon ECLIPSE E100). Dendrites in the bilateral hippocampus were traced from the cell soma to the projection terminus using the Neuron J plugin in ImageJ software. The Sholl analysis plug-in was employed for the analysis, and dendritic complexity was determined by the count of dendritic branch points at regular intervals from the cell bodies. The calculation of spine densities was performed on a single segment of dendrites longer than 10 μm in length from tertiary or higher-order apical and basal dendrites.

### 2.11 Enzyme-linked immunosorbent assay

The supernatant of the primary astrocyte culture medium and hippocampal tissues of mice were collected. The cholesterol content was investigated using the mouse total cholesterol enzyme-linked immunosorbent assay (ELISA) kits (JM-02912 M1, Jingmeibio, Yancheng, Jiangsu, China), following the manufacturer’s protocols. Protein concentrations of culture medium and hippocampal tissues were both assessed. The results were presented by cholesterol concentrations/protein concentrations.

### 2.12 Liquid chromatography–tandem mass spectrometry (LC/MS)

Serum (40 μL) was added into corresponding organic reagents according to the ratio of serum: methanol: acetonitrile (1:2:2, v/v/v), then vortexed fully for 2 min, and incubated at −20°C for 1 h. The mixtures were centrifuged at 13,000 rpm for 15 min at 4°C to precipitate the protein. The supernatant was lyophilized, and 100 μL 50% ACN was added for reconstitution, centrifuged, and tested on the machine. The hippocampus was immersed in 1.5 mL prechilled MeOH/H_2_O (−80°C, 1:1, v/v), and the mixtures were well ground. The mixtures were centrifuged at 14,000 g for 10 min at 4°C and then transferred approximately 1.2 mL of the supernatant to the clean tube. The supernatants were dried in a vacuum concentrator, and all the dried samples were reconstituted with 120 μL MeOH/H_2_O (1:1, v/v) solution for LC–MS analysis. Serum and hippocampal concentrations of TMAO were measured using liquid chromatograph coupled with a triple-quadrupole mass spectrometer (TSQ Quantis, Thermo Fisher). D9-TMAO was used as the internal standard.

### 2.13 MicroScale thermophoresis

The SREBP2 (544-1130aa) protein was selected with N-band trx tag for solubilization and C-band his tag for purification. It was constructed into pet32a vector, and *Escherichia coli* was used as the expression host to product purified SREBP2 proteins. Then, the purified SREBP2 proteins were labeled using Monolith Protein Labeling Kit RED-NHS (NanoTemper, Germany). TMAO was diluted with a 2-fold concentration gradient and incubated with 20 nM purified labeled SREBP2 protein. After loading the samples into NanoTemper glass capillaries, micro-thermophoresis was conducted with 80% light-emitting diode power and 80% MST. The equilibrium dissociation constant (KD) value was detected with the Monolith NT.115 (NanoTemper Technologies, Germany).

### 2.14 Molecular docking

TMAO 3D structure of SDF format was downloaded from PubChem data, optimized by ChemBio3D Ultra, and then converted into Protein Data Bank (PDB) format by using AutodockTools. The protein structure of mouse SREBP2 was downloaded from the UniProt database and was imported into AutoDockTools for hydrogenation, calculate charge, distribute charge, specify atomic type, and saved as PBD format. POCASA was applied to predict protein binding sites, and AutoDock Vina was used for docking. Finally, the interaction mode of the docking results was analyzed by PyMOL.

### 2.15 Statistical analysis

Statistical analyses were performed using GraphPad Prism 7.0. All values, except for Sholl interaction, are presented as mean ± SD. Normal distribution and variance homogeneity were assessed for each dataset. One-way analysis of variance (ANOVA) followed by Bonferroni’s or Turkey’s *post-hoc* tests was conducted to assess the statistical significance of variations among multiple groups. *P* values < 0.05 were considered statistically significant (*p < 0.05, **p < 0.01, ***p < 0.001, ****p < 0.0001).

## 3 Results

### 3.1 TMAO involves in memory impairment in the CSR mice

**Figure 1:**
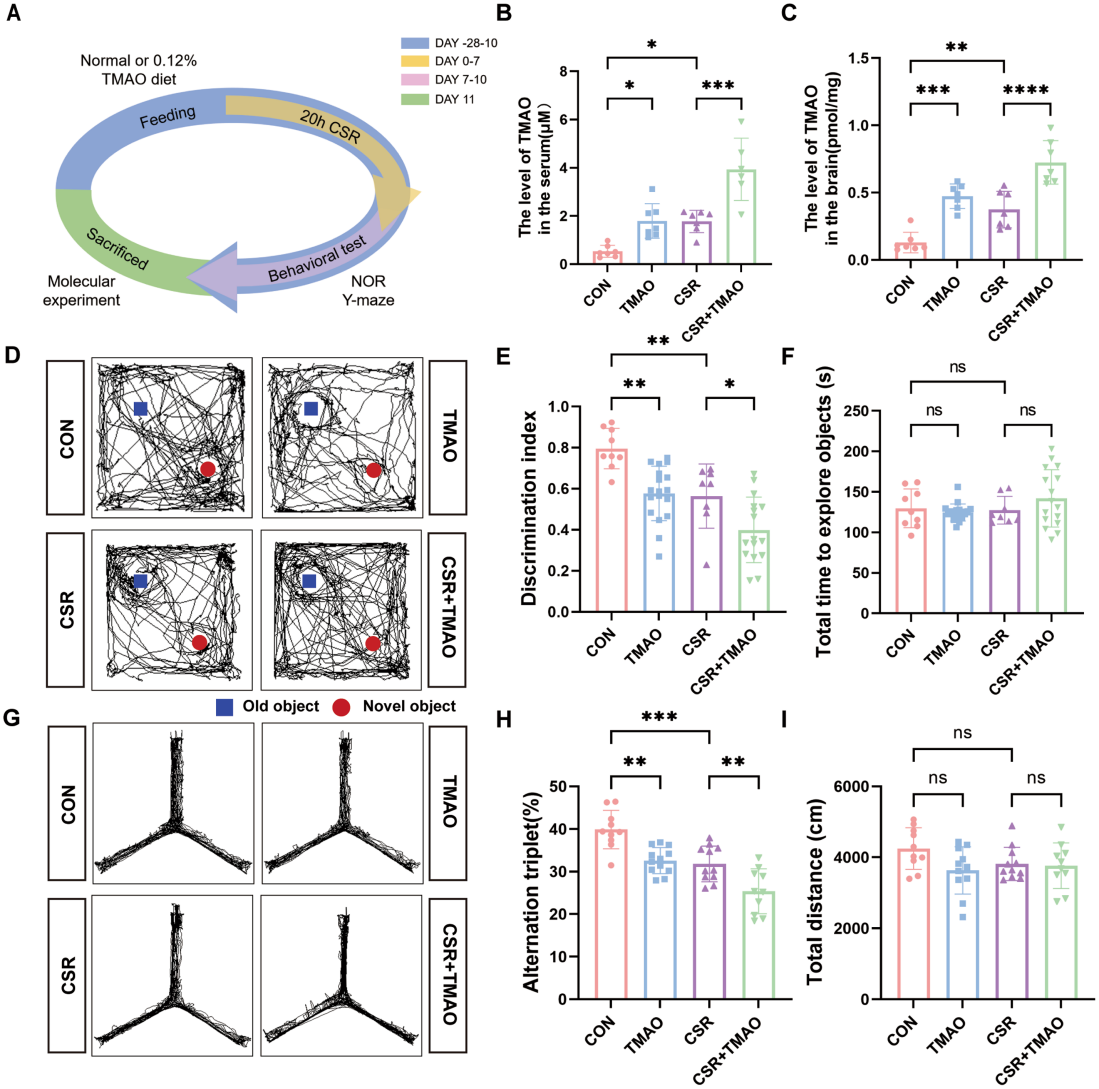
Elevated TMAO levels resulted in memory impairment in CSR mice. **(A)** Schematic diagram. **(B,C)** TMAO levels in the serum and hippocampus of four groups. **(D,E)** TMAO induces memory impairments in the NOR tests (*n* = 8–18 per group). **(F)** The total time to explore both objects in the NOR tests. **(G,H)** TMAO induces memory impairments in the Y-maze tests (*n* = 10–12 mice per group). **(I)** Total distance traveled in the Y-maze test. Data are represented as the mean ± SD.*p < 0.05, **p < 0.01, ***p < 0.001, and ****p < 0.0001, ns: no significance.

CSR mice were deprived of sleep for 20 h each day (from 8 to 4 pm the next day) for consecutive 1 week (Figure 1A). To explore whether TMAO is involved in CSR-related cognitive decline, we applied liquid chromatography–tandem mass spectrometry (LC/MS) to evaluate TMAO levels. As depicted in Figures 1B,C, the levels of TMAO in both the serum and brain were increased after CSR. To investigate the implication of TMAO on memory, we performed the NOR and Y-maze tests. In the NOR test, the CSR group and the TMAO group both spent much less time exploring a novel object than the control group (Figures 1D,E). At the same time, the CSR + TMAO mice spent a lower time exploring the novel object than the CSR mice (Figures 1D,E). However, there was no change in the total time to explore both objects in the four groups (Figure 1F).

In the Y-maze test, the spontaneous alternation was significantly reduced in the CSR mice and TMAO mice when compared to control mice (CON vs. CSR, *p* = 0.0006; CON vs. TMAO, *p* = 0.0016; Figures 1G,H) and was further decreased in the CSR + TMAO mice compared with CSR mice (*p* = 0.0074). The total distance showed no significant differences among the four groups (Figure 1I). Taken together, these results indicated that TMAO was involved in CSR-induced cognitive dysfunction.

### 3.2 TMAO involves in synapse loss and synaptic structural deficits in the CSR mice

**Figure 2:**
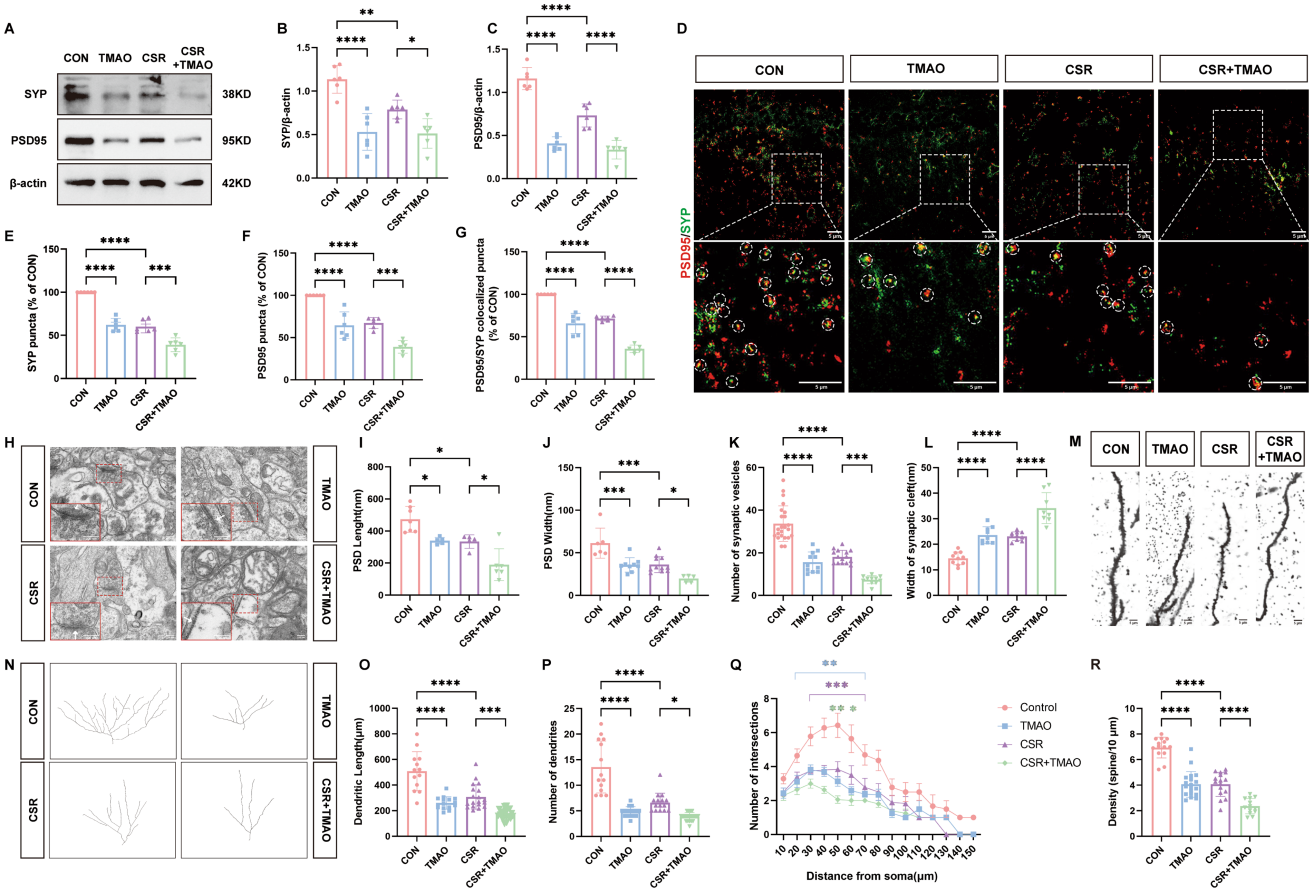
TMAO induced synapse loss and synaptic structural deficits in CSR mice. **(A)** Representative Western blot assay for the expression levels of SYP and PSD95 protein in four groups. **(B,C)** Quantification of the protein expressions of SYP and PSD95 (*n* = 6). **(D)** Representative images of immunofluorescence staining for PSD95 (red) and SYP (green) in the hippocampus. Scale bars = 5 μm. **(E–G)** Quantification of puncta in PSD95 and SYP and their colocalization in the hippocampus (*n* = 6). **(H)** Electron microscopy images of synapses in the hippocampus. Scale bars = 200 nm. **(I)** The length of the PSD (*n* = 5–7). **(J)** The width of the PSD (*n* = 5–11). **(K)** The number of synaptic vesicles (*n* = 11–23). **(L)** The width of the synaptic cleft (*n* = 8–10). **(M)** Representative images of Golgi-stained dendritic segments. Scale bars = 5 μm. **(N)** Representative images of neurons following Golgi staining and Sholl analysis. **(O,P)** Dendritic length and the number of dendrites for pyramidal neurons in the hippocampus of mice (*n* = 13–21). **(Q)** Dendritic complexity is shown by the number of dendritic intersections plotted against the distance from the soma in the hippocampal neurons (*n* = 14–20). Different asterisk colors show variations and the range of statistical significance. *(purple): CON vs. CSR; *(green): CSR vs. CSR + TMAO; *(blue): CON vs. TMAO. **(R)** Quantification of the dendritic spine density on pyramidal neuron dendrites in the hippocampus of mice (*n* = 13–19). Data are represented as the mean ± SD.*p < 0.05, **p < 0.01, ***p < 0.001, and ****p < 0.0001, ns: no significance.

We then assessed the expressions of SYP and PSD95 to explore the effect of TMAO on synaptic dysfunction. At the protein levels (Figure 2A), the expressions of SYP (CON vs. TMAO, *p* < 0.0001; CSR vs. CSR + TMAO, *p* = 0.0418; Figure 2B) and PSD95 (CON vs. TMAO, *p* < 0.0001; CSR vs. CSR + TMAO, *p* < 0.0001; Figure 2C) were both decreased in the mice supplemented with TMAO diet. In addition, the Western blotting results were corroborated by IF staining for SYP and PSD95 (Figures 2D–G). These data indicated that TMAO induced and aggravated synapse loss in the CSR mice.

Subsequently, the microscopy analysis results (Figure 2H) showed the reduction of PSD length (CON vs. TMAO, *p* = 0.0229; CSR vs. CSR + TMAO, *p* = 0.0168; Figure 2I) and width (CON vs. TMAO, *p* = 0.0006; CSR vs. CSR + TMAO, *p* = 0.0392; Figure 2J), as well as the number of presynaptic vesicles (CON vs. TMAO, *p* < 0.0001; CSR vs. CSR + TMAO, *p* = 0.0001; Figure 2K) were detected in the mice supplemented with TMAO diet. On the contrary, the TMAO-diet mice exhibited an increase in the width of the synaptic cleft (CON vs. TMAO, *p* < 0.0001; CSR vs. CSR + TMAO, *p* < 0.0001; Figure 2L).

We next investigated neural morphology by Golgi staining (Figures 2M,N). TMAO led to a significantly lower dendrite length (CON vs. TMAO, *p* < 0.0001; CSR vs. CSR + TMAO, *p* = 0.0001; Figure 2O) and lower number of dendrites (CON vs. TMAO, *p* < 0.0001; CSR vs. CSR + TMAO, *p* = 0.0129; Figure 2P). Furthermore, Sholl’s analysis revealed a significant reduction in the dendritic complexity of pyramidal neurons in the TMAO mice, which was aggravated in the CSR + TMAO mice (Figure 2Q). A significant reduction in the density of dendritic spines was also observed in the TMAO mice (CON vs. TMAO, *p* < 0.0001; Figure 2R) and decreased in CSR + TMAO mice (CSR vs. CSR + TMAO, *p* < 0.0001; Figure 2R). Collectively, these findings demonstrated that TMAO supplement diet related to synaptic proteins loss and dendritic morphology defects.

### 3.3 TMAO downregulates SREBP2 expression and inhibits cholesterol metabolism in the CSR mice

**Figure 3:**
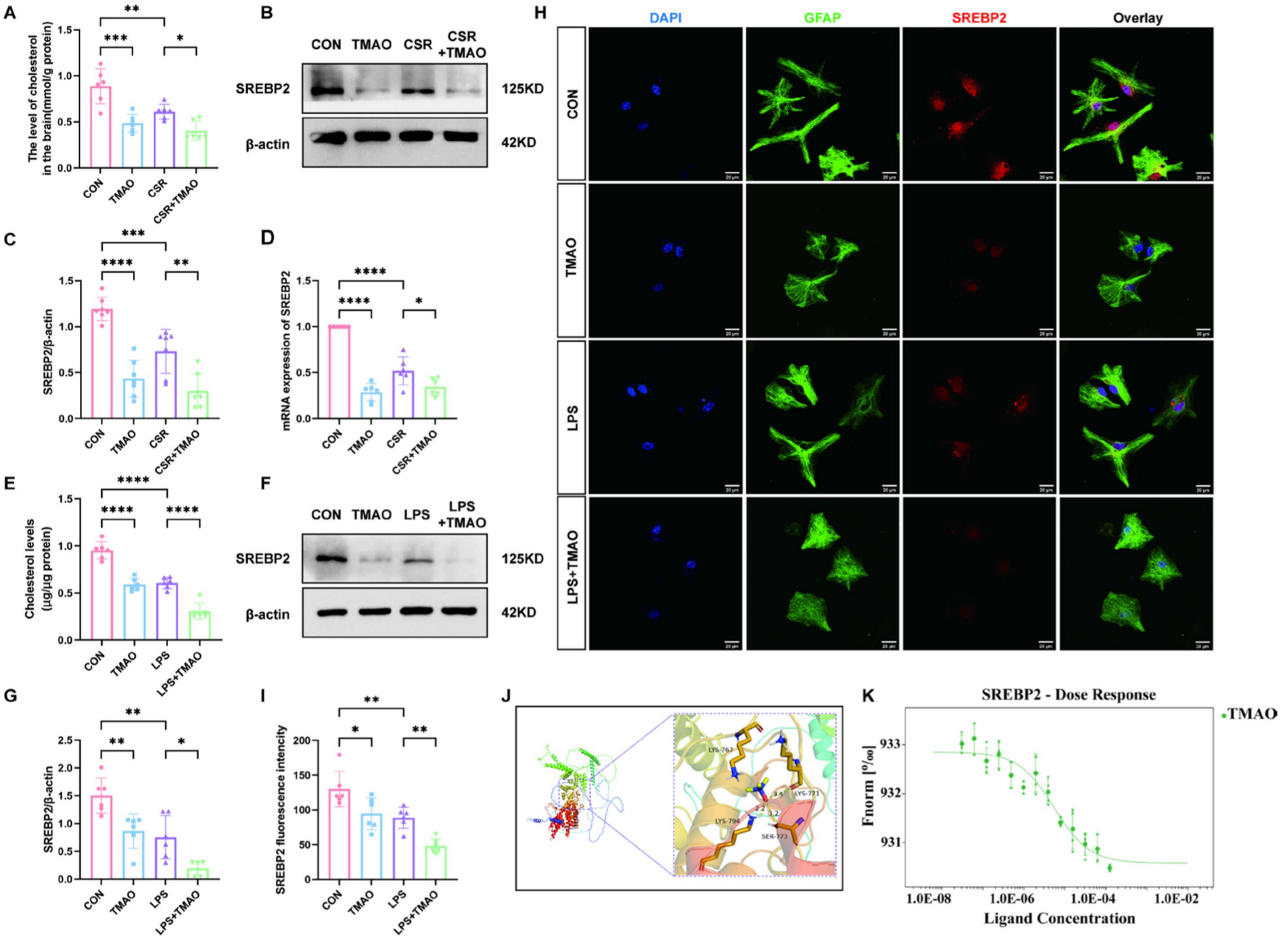
TMAO inhibited astrocytic cholesterol metabolism via reducing SREBP2 expression in CSR mice. **(A)** The level of cholesterol in the brain. **(B,C)** Western blot assay for the expression of SREBP2 protein in four groups. **(D)** PCR assay for SREBP2 mRNA levels. **(E)** The level of cholesterol in primary asvctrocyte culture medium. **(F,G)** Western blot assay for the expression levels of SREBP2 protein *in vitro*. **(H)** Co-localization of SREBP2 (red), GFAP (green), and DAPI (blue) was assessed in cultured astrocytes. **(I)** SREBP2 fluorescence intensity in four groups. **(J)** Molecular docking results showing the potential interaction between TMAO and SREBP2 protein. **(K)** MST results of TMAO and SREBP2. SREBP2 (20 nM) protein was incubated with increasing concentrations of TMAO. Data are represented as the mean ± SD. *p < 0.05, **p < 0.01, ***p < 0.001, and ****p < 0.0001, ns: no significance. (*n* = 6–7 per group).

To evaluate the impact of CSR on cholesterol metabolism, we examined the hippocampal cholesterol content and the expression of its major transcription factor SREBP2. We found that CSR mice displayed a notable reduction in brain cholesterol content (Figure 3A). Furthermore, Western blotting showed that SREBP2 was reduced in CSR mice (Figures 3B,C). Similarly, the qPCR result was consistent with Western blotting (Figure 3D). In addition, qPCR results of HMGCR, a downstream of SREBP2, showed that HMGCR reduced in CSR mice, which indicated that the activity of SREBP2 decreased (Supplementary Figure S1A). To further evaluate whether elevated concentrations of TMAO in serum and brain induced cholesterol deficits, mice were supplemented with 0.12% TMAO. We detected that TMAO caused suppression of cholesterol levels (CON vs. TMAO, *p* < 0.0001; CSR vs. CSR + TMAO, *p* = 0.0480; Figure 3A). As expected, TMAO reduced the expression of SREBP2 at the protein level (CON vs. TMAO, *p* < 0.0001; CSR vs. CSR + TMAO, *p* = 0.0018; Figures 3B,C) and mRNA level (Figure 3D).

To verify that TMAO-induced reduction in brain cholesterol level is because of the reduction in cholesterol level in astrocytes, we performed *in vitro* experiments. We stimulate primary astrocytes with 0, 50 μM, 100 μM, 200 μM, or 400 μM TMAO. The Western blotting analysis showed a substantial decrease in astrocytic SREBP2 protein expression following treated with 100–400 μM TMAO (*p* < 0.05; Supplementary Figure S2A) and the SREBP2 expression showed no significant differences among these three groups (*p* > 0.05; Supplementary Figure S2B). Accordingly, TMAO (100 μM) and LPS (100 ng/mL) were used to stimulate primary astrocytes. The cholesterol concentration in the culture medium revealed that cholesterol concentration was remarkably lower in the TMAO group than in the CON group (*p* < 0.0001; Figure 3E), which was aggravated by LPS (LPS vs. LPS + TMAO, *p* < 0.0001; Figure 3E). As depicted in Figures 3F,G, SREBP2 expression was decreased in the TMAO group compared to the CON group (*p* = 0.0080) and was further decreased in the LPS + TMAO group (LPS vs. LPS + TMAO, *p* = 0.0213). Immunofluorescence results were consistent with Western blotting analysis (Figures 3H,I). Above all, we confirmed that TMAO downregulated cholesterol metabolism by inhibiting SREBP2 expression in CSR mice.

Subsequently, we assessed the relationship between TMAO and SREBP2 by molecular docking and MST experiment. The molecular docking predicted results showed that TMAO could form a critical hydrogen bond and hydrophobic force with SREBP2 (Figure 3J), which is the key to the interaction. The result of mean binding energies of docking for the TMAO with SREBP2 was −5.8 kcal/mol, proving that it has a better binding effect. MST is a method that labeled SREBP2 protein with fluorescence and required increasing concentrations of TMAO with an established quantity of SREBP2 protein to determine KD values in solution. TMAO binding to SREBP2 protein yielded a KD value of 5.44 ± 1.89 μM, which belongs to strong binding in the field of protein-small molecule binding (Figure 3K).

### 3.4 DIM improves astrocytic cholesterol deficits by reducing TMAO expression in CSR mice

**Figure 4:**
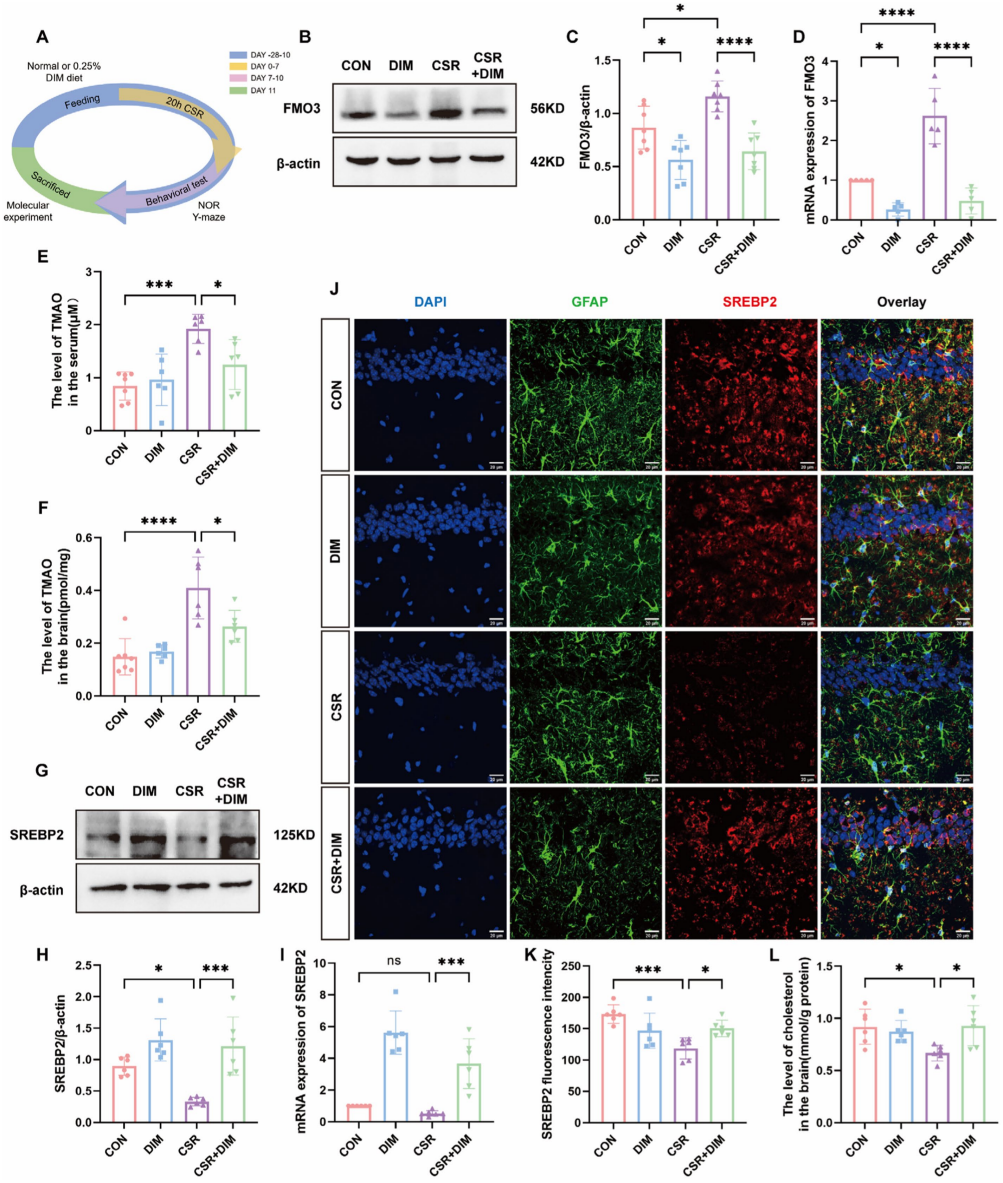
DIM suppressed TMAO expression and improved cholesterol synthesis in astrocytes. **(A)** Schematic diagram. **(B)** Representative Western blots of FMO3 protein in four groups. **(C)** Quantification of the protein expressions of FMO3. **(D)** PCR assay for FMO3 mRNA levels. **(E,F)** TMAO levels in the serum and hippocampus of four groups. **(G)** Representative Western blot assay for the expression levels of SREBP2 protein in four groups. **(H)** Quantification of the protein expressions of SREBP2. **(I)** PCR assay for SREBP2 mRNA levels. **(J)** Representative images of immunofluorescence staining for SREBP2 (red) and its colocalization with GFAP (green) in the hippocampus of four groups. Scale bars = 20 μm. **(K)** SREBP2 fluorescence intensity in four groups. **(L)** The level of cholesterol in the brain. Data are represented as the mean ± SD.*p < 0.05, **p < 0.01, ***p < 0.001, and ****p < 0.0001, ns: no significance. (*n* = 5–7 per group).

Figure 4A shows the experimental process of DIM intervention. To determine the cause of elevated TMAO levels in the CSR mice, Western blotting was applied to evaluate hepatic FMO3 expression level. The results showed that hepatic FMO3 was significantly increased in CSR mice (Figures 4B,C). Consistently, qPCR confirmed a further increased expression of FMO3 mRNA level in CSR mice (Figure 4D). Accordingly, the mice were treated with the FMO3 inhibitor 3,3′-diindolylmethane (DIM; Cashman et al., 1999; Katchamart et al., 2000). Mice were placed on a diet with or without 0.25% DIM supplementation for 4 weeks (Chen et al., 2019). As expected, DIM inhibited hepatic FMO3 protein level in the CSR mice (CSR vs. CSR + DIM, *p* < 0.0001; Figures 4B,C) as well as mRNA level (Figure 4D). Meanwhile, serum and hippocampal TMAO concentrations were decreased after DIM treatment (CSR vs. CSR + DIM: serum, *p* = 0.0292; brain, *p* = 0.0234; Figures 4E,F).

In contrast, Western blotting results showed that the SREBP2 protein expression was significantly increased in the CSR + DIM group than in the CSR group (Figures 4G,H). The mRNA level of SREBP was consistent with Western blotting (Figure 4I). Immunofluorescence results were consistent with Western blotting and PCR analysis (Figures 4J,K). Consistently, brain cholesterol concentrations of CSR mice were also increased following DIM treatment (Figure 4L). Overall, these results confirmed that DIM improved CSR-induced astrocytic cholesterol deficits through TMAO downregulation.

### 3.5 DIM reverses CSR-induced synapse loss and synaptic structural deficits

**Figure 5:**
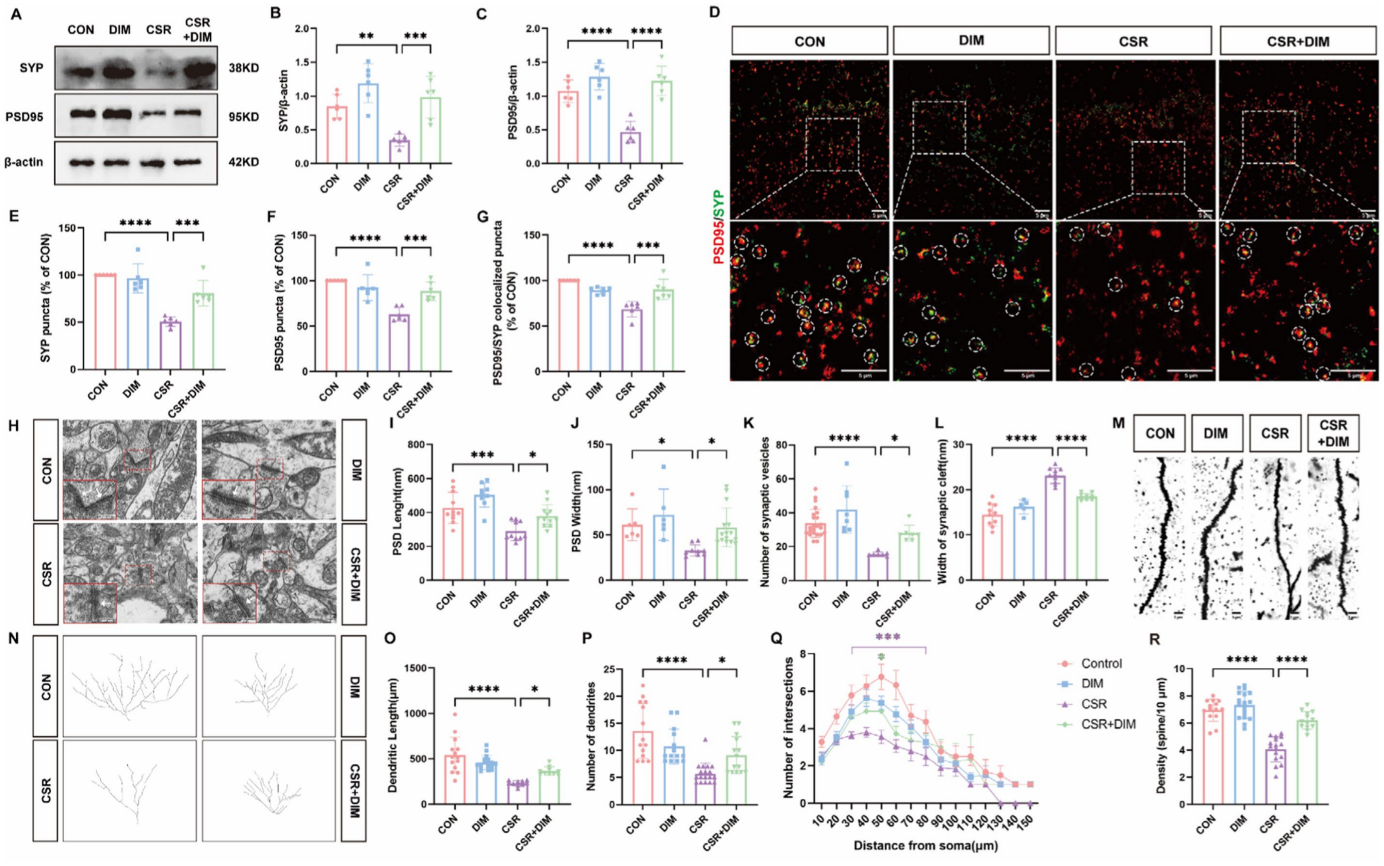
DIM treatment reversed synapse loss and synaptic structural deficits in CSR mice. **(A)** Representative Western blot assay for the expression levels of SYP and PSD95 protein in four groups. **(B,C)** Quantification of the protein expressions of SYP and PSD95 (*n* = 6). **(D)** Representative images of immunofluorescence staining for PSD95 (red) and SYP (green) in the hippocampus. Scale bars = 5 μm. **(E–G)** Quantification of puncta in PSD95 and SYP and their colocalization in the hippocampus (*n* = 6). **(H)** Electron microscopy images of synapses in the hippocampus of four groups. Scale bars = 200 nm. **(I)** The length of the PSD (*n* = 9–12). **(J)** The width of the PSD (*n* = 6–15). **(K)** The number of synaptic vesicles (*n* = 7–23). **(L)** The width of the synaptic cleft (*n* = 5–10). **(M)** Representative images of Golgi-stained dendritic segments. Scale bars = 5 μm. **(N)** Representative images of neurons following Golgi staining and Sholl analysis. **(O,P)** Dendritic length and the number of dendrites for pyramidal neurons in the hippocampus of mice (*n* = 10–17). **(Q)** Dendritic complexity is shown by the number of dendritic intersections plotted against the distance from the soma in the hippocampal neurons (*n* = 14–18). Different asterisk colors show variations and the range of statistical significance. *(purple): CON vs. CSR; *(green): CSR vs. CSR + DIM. **(R)** Quantification of the dendritic spine density on pyramidal neuron dendrites in the hippocampus of mice (*n* = 13–18). Data are represented as the mean ± SD.*p < 0.05, **p < 0.01, ***p < 0.001, and ****p < 0.0001, ns: no significance.

To investigate the impact of DIM on synaptic dysfunction induced by CSR, we assessed the expression of SYP and PSD95 in CSR + DIM mice. At the protein levels (Figure 5A), DIM dramatically improved CSR-induced reduction in SYP (CSR vs. CSR + DIM, *p* = 0.0007; Figure 5B) and PSD95 (*p* < 0.0001; Figure 5C). In addition, immunofluorescence staining results showed that the quantification of puncta in PSD95 and SYP and their colocalization showed a considerable loss of synapses in CSR mice (Figures 5D–G), while SYP and PSD95 puncta and colocalized puncta were restored in the CSR + DIM mice compared with CSR mice (Figures 5D–G). These findings suggested that DIM alleviated synapse loss after CSR.

Moreover, electron microscopy analysis was utilized (Figure 5H). In CSR mice, PSD length (Figure 5I) and width (Figure 5J) and the number of presynaptic vesicles (Figure 5K) were all reduced compared with the CON group. On the contrary, there was an observed increase in the width of the synaptic cleft in CSR mice (Figure 5L). Nevertheless, DIM was found to reverse these alterations in synaptic ultrastructure (CSR vs. CSR + DIM; Figures 5I–L).

We next investigated neural morphology by Golgi staining (Figures 5M,N). The CSR group exhibited a statistically significant reduction in dendrite length (Figure 5O) and dendrite number (Figure 5P) of hippocampal pyramidal neurons compared to the control group, which were partly reversed by DIM (Figures 5O,P). Moreover, compared with control mice, the Sholl intersections were fewer in pyramidal neurons of CSR mice at a distance of 30–80 μm from soma to the terminal (Figure 5Q), which were also partly rescued by DIM (CSR vs. CSR + DIM: 50 μm, *p* = 0.0248), suggesting that DIM mildly enhanced dendrite complexity of CSR mice. In parallel, a significant decrease in the density of dendritic spines was detected in the CSR mice (Figure 5R) but markedly increased in CSR + DIM mice (CSR vs. CSR + DIM: *p* < 0.0001). Taken together, these findings demonstrated that DIM reversed the depletion of synapse-related proteins and dendritic morphological abnormalities in CSR mice.

### 3.6 DIM alleviates memory impairment induced by CSR

**Figure 6:**
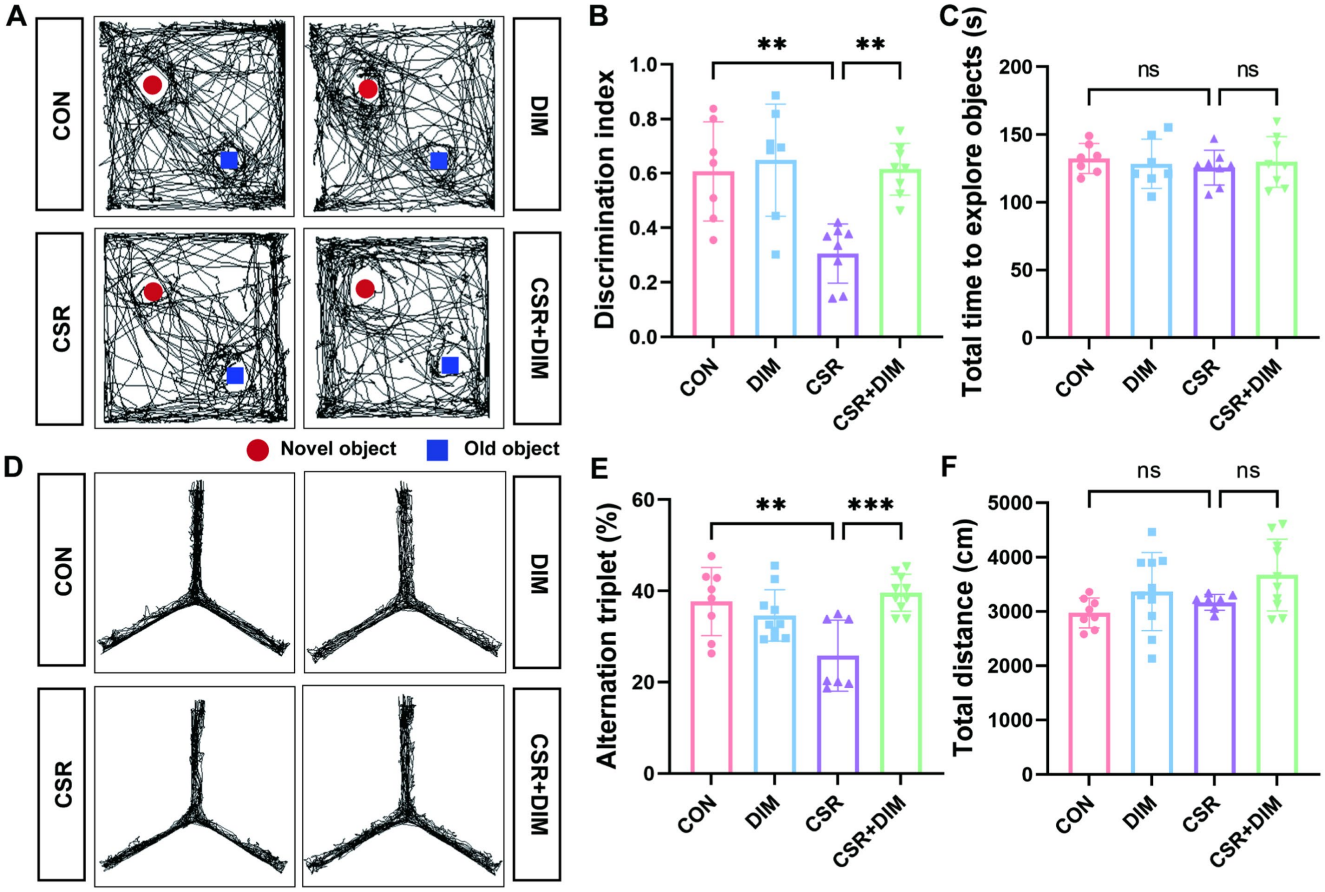
DIM treatment mitigated learning and memory impairments in CSR mice. **(A,B)** DIM treatment improved mice performance in the NOR test. **(C)** The total time to explore both objects in the NOR test. **(D,E)** DIM treatment improved mice performance in the Y-maze test. **(F)** The total distance traveled in the Y-maze test. Data are represented as the mean ± SD.*p < 0.05, **p < 0.01, ***p < 0.001, and ****p < 0.0001, ns: no significance. (*n* = 7–10 per group).

To assess the effect of DIM on memory in CSR mice, we performed NOR and Y-maze tests to evaluate the ability of mice. In the NOR test, the CSR + DIM mice explored the novel object for a longer period of time compared to the CSR mice (Figures 6A,B). However, the total time to explore both objects was not different among the four groups (*p* > 0.05; Figure 6C). In the Y-maze test, the spontaneous alternation was increased in the CSR + DIM mice than in the CSR mice (Figures 6D,E). The total distance showed no significant differences among the four groups (*p* > 0.05; Figure 6F). These results indicated that DIM effectively attenuated CSR-induced memory deficits.

## 4 Discussion

**Figure 7:**
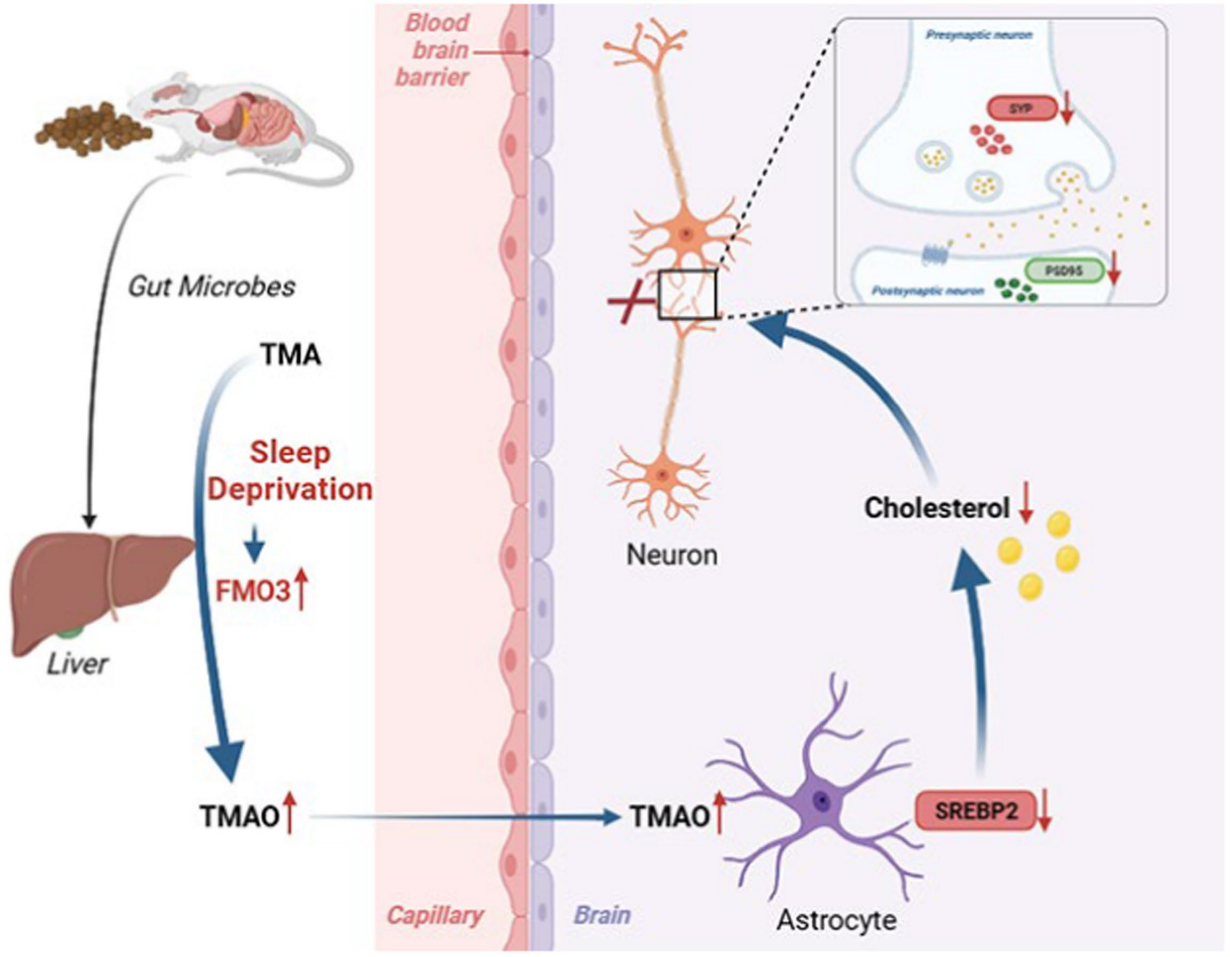
Working model illustrating that sleep deprivation elevated FMO3/TMAO levels and then decreased astrocyte SREBP2 expression and cholesterol synthesis, which leading to structural and functional synaptic deficits and cognitive deficits.

In the current study, we first demonstrated that TMAO was involved in SD-induced cognitive dysfunction and FMO3 inhibitor DIM alleviated cognitive impairment in CSR mice by reducing TMAO level, upregulating astrocytic SREBP2 expression and brain cholesterol content, and finally reversing a synaptic loss, which offers novel insight into therapies for cognitive impairment after sleep deprivation (Figure 7). Indeed, the FMO3 inhibitor employed in this study, DIM, is already available as nutritional supplements (Williams et al., 2001), which may have a great deal of promise for preventing cognitive impairment induced by sleep loss. Moreover, our study provided evidence for the first time that TMAO inhibited astrocytic SREBP2 expression and interacted with it, offering a supplementary discovery of TMAO.

The liver–brain axis serves as an intricate communication network that connects the liver and the central nervous system (CNS). Increased levels of TMAO, one of the FMO3 metabolites, have been demonstrated highly associated with cognitive impairment in many diseases, including diabetes (Lanz et al., 2022), brain aging (Brunt et al., 2021), and neurodegenerative disorders such as AD (Vogt et al., 2018), PD (Chung et al., 2021), and HD (Borwankar et al., 2011). It has been demonstrated that TMAO could penetrate the BBB (Del Rio et al., 2017), which indicated that increased TMAO in the CNS may accelerate cognitive impairment. Consistent with these studies, our data first suggested that the concentrations of TMAO in both the serum and brain were increased because of the increased FMO3 level after CSR. In addition, elevated TMAO in the serum and hippocampus, due to dietary supplements, aggravated CSR-induced cognitive deficits. In our behavioral experiment, the experimental results of the Y-maze test had obvious statistical differences, and the results were relatively stable. In the NOR experiment, the results of the CON and CSR groups were relatively stable, but the results of the TMAO group and the CSR + TMAO group were not very stable (greater within-group differences). To make the results more accurate and minimize the sacrifice of mice, we repeated NOR with increased number of mice in the TMAO and CSR + TMAO groups to obtain more accurate experimental results, which explained why the groups were unbalanced in Figure 1E. Our above findings were consistent with a recent study conducted by Li et al. (2018), which revealed elevated TMAO levels induced age-related cognitive dysfunction and TMAO supplements aggravated this impairment. On the contrary, it has also been reported that TMAO has a positive effect on BBB’s integrity and cognitive ability (Hoyles et al., 2021). The possible reason for this difference is due to different concentrations of TMAO. As aging is associated with cognitive decline, in this study, we used 9- to 10-week-old mice to build the CSR model and conducted behavioral tests at 10- to 11-week-old mice to exclude the influence of age on the experimental results. In the study of Hoyles et al. (2021), a physiological concentration of TMAO was used for long-term intervention, and their study also showed that a high concentration of TMAO intervention could increase cell permeability *in vitro*, suggesting different concentrations of TMAO may have different effects on cognitive function. Chronic exposure to TMAO increased the risk for chronic inflammatory malignancies such as colorectal cancer (Bae et al., 2014). However, transiently increased TMAO promoted immunotherapy of pancreatic cancer, suggesting that strategies that acutely increase TMAO may be a reasonable intervention (Mirji et al., 2022). Moreover, decreased TMAO levels showed a clear improvement in cognitive alterations (Brunt et al., 2020). Similarly, we confirmed that mice supplemented with the DIM diet showed lower FMO3/TMAO levels and ameliorated CSR-induced memory impairment, indicating that DIM is expected to be a therapeutic drug for CSR-induced cognitive impairment.

FMO3/TMAO pathway is related to peripheral cholesterol metabolism (Warrier et al., 2015). Research findings have demonstrated that the incorporation of TMAO as a dietary supplement resulted in a reduction in reverse cholesterol transport in the peripheral system (Janeiro et al., 2018). Reverse cholesterol transport refers to the process by which cholesterol present in the arterial wall is transported to the liver through high-density lipoproteins (HDLs), subsequently being excreted into bile and ultimately eliminated from the body via fecal excretion (Dietschy and Turley, 2002; Rader et al., 2009). Of note, Cyp7a1, the primary bile acid synthase enzyme involved in the catabolism of cholesterol, was reduced in expression as a result of TMAO. The effect of TMAO was found to be linked with diminished synthesis and secretion of bile acids and heightened cholesterol accumulation (Koeth et al., 2013). Nevertheless, no investigations have explored the correlation between TMAO and cerebral cholesterol metabolism. In our study, we first confirmed that TMAO inhibited astrocytic SREBP2 expression and cholesterol level *in vitro* and *in vivo* experiments, which was significantly reversed by DIM treatment. This finding is inconsistent with the previous report on peripheral systems, which reported TMAO reduced reverse cholesterol transport (Janeiro et al., 2018). One explanation may be that plasma cholesterol and intracerebral cholesterol are two independent systems, and reverse cholesterol transport is one of the cholesterol important metabolic pathways in the peripheral system but conversion to oxysterol by cholesterol 24-hydroxylase is the cholesterol major excretion way in the brain. In our study, we observed that TMAO downregulated both the transcription and protein levels of SREBP2 and the possibility of direct interaction between TMAO and SREBP2. Hence, we speculate that TMAO may affect SREBP2 from three aspects: transcriptional level, protein level, and biological function. First, our data showed that TMAO reduced SREBP2 mRNA levels. Second, given study has confirmed that ChREBP promoted SREBP2 degradation by facilitating its ubiquitination (Zhang et al., 2017). It remains to be determined whether TMAO enhances SREBP2 ubiquitination through direct binding. Of course, we acknowledge that changes in protein levels may also be solely due to a decrease in transcription levels. Third, the interaction of TMAO and SREBP2 may influence the nuclear localization of SREBP2, which is the active form of it. Above all, the specific effect of TMAO on SREBP2 needs to be further explored in our future experiments.

Brain cholesterol metabolism is locally synthesized because of the BBB and cholesterol in the adult brain is mainly synthesized in astrocytes (Li et al., 2022). The modulation of cholesterol supply to neurons by astrocytic cholesterol metabolism plays a crucial role in cognitive processes such as learning and memory in the brain. Cholesterol level was severely hindered in models of diabetes and Huntington’s disease, as a result of the downregulation of numerous genes involved in the cholesterol biosynthesis pathway, including SREBP2 (Suzuki et al., 2010; Birolini et al., 2021). This is consistent with this study in that we first found a reduction in astrocytic SREBP2 and cholesterol content in the CSR mice. Interestingly, brain cholesterol turnover was increased in Alzheimer’s and has been linked to the pathogenesis of AD (Wang et al., 2021). This difference may be explained by the fact that cholesterol homeostasis is a dynamic equilibrium process and is vital for brain function. Cholesterol excessive accumulation resulted in beta-amyloid and decreased cholesterol synthesis led to synaptic defects. Epidemiological data indicated a correlation between elevated serum cholesterol and diabetes and prolonged sleep deprivation in humans which is contrary to the results in the brain (Semova et al., 2020; Aho et al., 2016). This apparent contradiction can be elucidated by the presence of the BBB, which establishes plasma cholesterol and intracerebral cholesterol as distinct systems. Consequently, the increase in plasma cholesterol levels has no effect on intracerebral cholesterol levels. In addition, many studies have begun to pay attention to cholesterol metabolism in microglia (Tcw et al., 2022; Nugent et al., 2020), and our previous study has revealed that microglial activation is excessively enhanced in the CSR mice (Tan et al., 2023). However, whether cholesterol metabolism in microglia is involved in CSR-induced cognitive impairment has not been clarified. Further investigations are required to clarify the detailed mechanisms.

Previous studies have suggested that brain cholesterol metabolism showed a strong correlation with synapse formation, and disruption of the astrocytic cholesterol synthesis pathway can lead to axonal growth restriction, synaptic damage, and memory impairment (Ferris et al., 2017; Xu et al., 2022; Santello et al., 2019). Consistent with previous studies (Li X. et al., 2023), our study indicated that CSR mice exhibited impaired working memory, accompanied by a reduction in synaptic density and dendritic spines. In line with the decreased expression of the synaptic markers PSD95 and SYP, we found that hippocampus pyramidal neurons in CSR mice had considerably shorter dendritic lengths and connections. Ferris et al. (2017) revealed that when the cholesterol synthesis decreased in astrocytes, the cholesterol synthesis of neurons increased, which proved the compensatory effect of cholesterol synthesis of neurons, but we still found aberrations in neuronal morphology in CSR mice. The possible reasons are as follows: The neurons and astrocytes in the brain have the ability to synthesize cholesterol. However, in adult, brain cholesterol is predominantly synthesized in glial cells, primarily astrocytes (Berghoff et al., 2021; Fünfschilling et al., 2012). Although Ferris et al. revealed the compensatory effect of cholesterol synthesis of neurons, they also found that loss of SREBP2 from glia inhibited neurite outgrowth, certificating that the compensatory ability to synthesize cholesterol of neurons is limited (Ferris et al., 2017). Therefore, despite the expected cholesterol compensatory mechanism by neurons, we still found aberrations in neuronal morphology in CSR mice. In addition, we found TMAO-induced and aggravated synapse loss and synaptic structural deficits in CSR mice. DIM ameliorated cognitive deficits by improving structural and functional synaptic impairments which were induced by elevated TMAO levels in CSR mice.

There were a few limitations in this study. First, this study confirmed that DIM plays an important role in alleviating CSR-induced cognitive impairment by upregulating astrocytic cholesterol content. However, we cannot exclude the possibility that DIM may attenuate cognitive deficits by acting on other aspects, such as neuroinflammation and astrocyte activation. Therefore, we are currently constructing glial SREBP2 over-expression mice to provide direct evidence. Second, we have not investigated the metabolic pathway of how TMAO promotes SREBP2 decomposition to reduce its level after direct interaction in CSR mice and the mechanism remains to be explored further in the future. Third, TMAO is derived from the catabolism of dietary nutrients by the gut microbiota, but concerning the complex mechanisms of gut microbiota influencing cognitive decline, we have not investigated whether CSR can affect the changes of some flora or TMA and then affect TMAO in this study, which requires further research. Finally, the male mice were only used as the studied subjects in the current study and whether our findings can be extrapolated to females remains unknown and warrants further study.

In conclusion, our study revealed that CSR-elevated FMO3/TMAO levels with the subsequent decreased astrocytic SREBP2 expression and cholesterol content can lead to synaptic structural and functional deficits as well as cognitive deficiency. Therefore, pharmacological therapies that lead to decreased TMAO levels, such as DIM, could open a new avenue by targeting the liver–brain axis for the future treatment of cognitive impairment after sleep deprivation.

## Data Availability

The original contributions presented in the study are included in the article/Supplementary material, further inquiries can be directed to the corresponding authors.
