# Supplementary material for: TMAO is involved in sleep deprivation-induced cognitive dysfunction through regulating astrocytic cholesterol metabolism via SREBP2
Source: Front Mol Neurosci. 2024 Nov 28;17:1499591. doi: 10.3389/fnmol.2024.1499591 (PMC11634841; doi:10.3389/fnmol.2024.1499591)
Supplement: Supplementary file 1 [file Table_1.DOCX]

**Supplemental Digital Content**

**
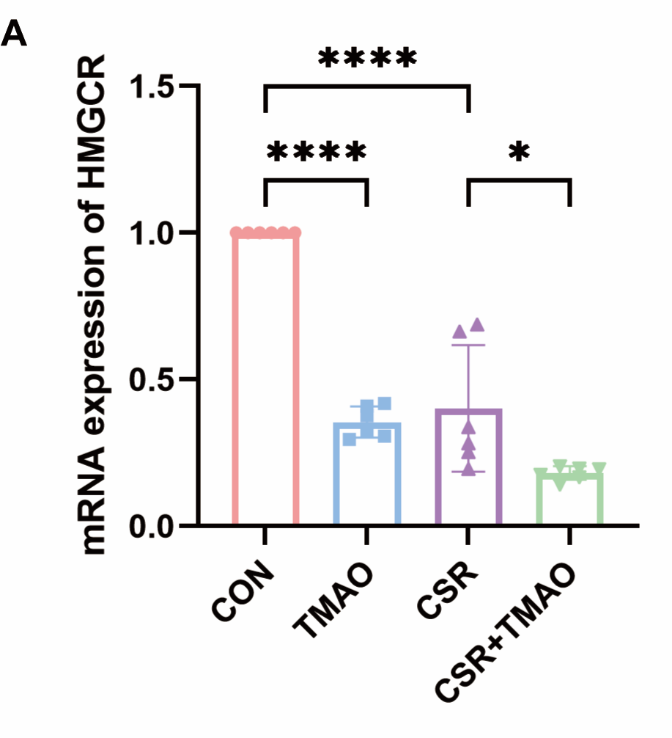
**

**FIGURE S1**

Effect of TMAO intervention on HMGCR expression.

(A) PCR assay for HMGCR mRNA levels.


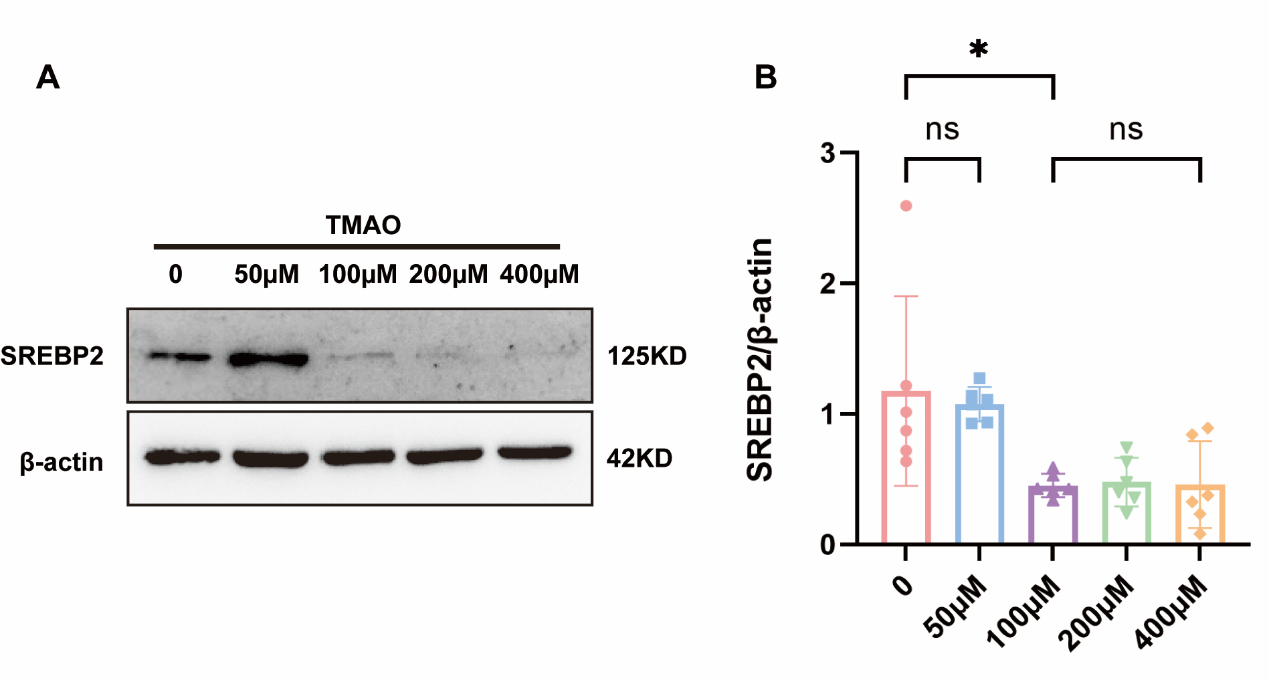


**FIGURE S2**

Effect of TMAO intervention at different concentrations on SREBP2 expression.

(A, B) Western blotting results showed that the protein level of astrocyte SREBP2 treated with 0-400μM TMAO.
